# Expression Pattern of *FT/TFL1* and miR156-Targeted *SPL* Genes Associated with Developmental Stages in *Dendrobium catenatum*

**DOI:** 10.3390/ijms20112725

**Published:** 2019-06-03

**Authors:** Jie Zheng, Yuru Ma, Mengyao Zhang, Meiling Lyu, Yuan Yuan, Binghua Wu

**Affiliations:** Fujian Provincial Key Laboratory of Plant Functional Biology, College of Horticulture, Fujian A & University, Fuzhou 350002, China; zhengjie_stephy@sina.com (J.Z.); mayuru916@gmail.com (Y.M.); zmengy11@163.com (M.Z.); mllv2009@126.com (M.L.); yuanyuan@fafu.edu.cn (Y.Y.)

**Keywords:** *Dendrobium catenatum*, flowering, juvenile, phosphatidylethanolamine-binding protein (PEBP) family, *TFL1*-like, miR156, squamosa promoter binding protein-like (SPL) transcription factor

## Abstract

Time to flower, a process either referring to juvenile–adult phase change or vegetative–reproductive transition, is strictly controlled by an intricate regulatory network involving at least both *FT/TFL1* and the micro RNA (miR)156-regulated *SPL* family members. Despite substantial progresses recently achieved in *Arabidopsis* and other plant species, information regarding the involvement of these genes during orchid development and flowering competence is still limited. *Dendrobium catenatum*, a popular orchid species, exhibits a juvenile phase of at least three years. Here, through whole-genome mining and whole-family expression profiling, we analyzed the homologous genes of *FT/TFL1*, miR156, and *SPL* with special reference to the developmental stages. The *FT/TFL1* family contains nine members; among them, *DcHd3b* transcribes abundantly in young and juvenile tissues but not in adult, contrasting with the low levels of others. We also found that mature miR156, encoded by a single locus, accumulated in large quantity in protocorms and declined by seedling development, coincident with an increase in transcripts of three of its targeted *SPL* members, namely *DcSPL14*, *DcSPL7*, and *DcSPL18*. Moreover, among the seven predicted miR156-targeted *SPLs*, only *DcSPL3* was significantly expressed in adult plants and was associated with plant maturation. Our results might suggest that the juvenile phase change or maturation in this orchid plant likely involves both the repressive action of a *TFL1*-like pathway and the promotive effect from an *SPL3*-mediated mechanism.

## 1. Introduction

Flowering, the reproductive process of higher plants, is an important trait vital to agriculture practice. The timing of flower initiation is determined by measuring the day-length (photoperiod) and/or low temperature (vernalization) in many annual plants, including the model plants *Arabidopsis* and rice [1]. These annual plants may acquire competence to flower shortly after seed germination. However, perennials, especially woody trees, take longer periods, lasting from a few weeks to many years for vegetative growth in order to achieve flowering, even under favorable conditions, a prolonged stage called juvenile phase [1,2]. 

The *Arabidopsis* flowering locus T (*FT*) [3] and its rice ortholog *Hd3a* [4] induce flowering in a photoperiod-dependent manner, whereas their homolog terminal flower 1 (*TFL1*) [5] from the same phosphatidylethanolamine-binding protein (PEBP) gene family delays flowering. These proteins are mainly expressed in leaves and move to the shoot apices to activate or repress floral bud development in response to environmental and developmental cues including day-length, cold signals, and maturity status; hence, the molecular identity as the long-sought florigen and anti-florigen is assigned [6,7,8,9]. The *FT/TFL1*-mediated photoperiod flowering pathway is widespread in angiosperm species [6,10], and homologous members of the family also exist in gymnosperms where they seem to be involved in regulation of growth rhythm, bud dormancy, and reproductive development process [10]. The *FT*/*TFL1* family can be divided into three subfamilies: *FT*-like, *TFL1*-like, and *MFT*-like. The *MFT (*mother of *FT* and *TFL1*) homologs were identified in genomes of some bryophytes, which lack the other *FT*/*TFL1*-like genes [11]. *MFT*-like genes, together with mixed *FT/TFL1*-genes, also present in gymnosperm species [11,12]. It is, therefore, suggested that *MFT*-like genes are ancestral to both *FT*-like and *TFL1*-like genes and that the separated functions of *FT* and *TFL1* were evolutionarily attained after divergence of gymnosperms and angiosperms (see Reference [6] for a review). Works in *Arabidopsis* reveal that, at the shoot apex, the mobile *FT* forms a complex with both a 14-3-3 protein (a G-box containing factor) [13] and a bZIP transcription factor FD which is apex-specific. This FT/FD/14-3-3 complex activates several MADS box genes or floral meristem identity genes, including Apetala 1 (*AP1*), Fruitfull (*FUL*), suppressor of overexpression of Constans 1 (*SOC1*) and the plant-specific transcription factor Leafy (*LFY*) to initiate vegetative to floral transition [8,14,15,16]. The antagonistic TFL1 proteins also interact competitively with FD and 14-3-3 protein to repress downstream genes for flowering [13,17], although this opposing mechanism in controlling flowering time is still not fully explained [9,10,18,19].

In parallel to and independent of the *FT*/*TFL1* pathway, several of the miRNA156-regulated squamosa promoter binding protein-like (SPL) factors that specifically expressed at the shoot apex also act upstream of some of these MADS-box-containing floral regulators, albeit in an age-dependent manner [20,21,22]. MiR156 belongs to a group of microRNAs (miRNAs) that function as negative regulators for expression of protein-coding genes, by transcript cleavage and translational inhibition upon base-pairing to their targeted messenger RNAs (mRNAs) [23,24,25]. MicroRNAs are short non-coding eukaryotic RNAs, typically 20–24 nt, which are derived from pri-miRNAs through the action of the conserved small RNA machinery [26]. In *Arabidopsis*, mature miR156 may be produced from eight precursor genes, namely miR156A through miR156H. By targeting the expression of several *SPL* genes, miR156 plays pivotal roles in vegetative phase change and shoot development as first demonstrated in *Arabidopsis*, e.g., overexpression of miR156 led to a prolonged juvenile to adult phase [27,28,29]. 

An age-associated reduction in the abundance of miR156 and the resultant increased levels in its target *SPL* mRNAs are necessary to induce vegetative phase change and flowering in *Arabidopsis*, maize, perennial *A. alpina*, and Brassicaceae species [21,22,27,28,30]. Among the ten *SPL* genes that are targeted by miR156 in *Arabidopsis*, *SPL9/SPL15* and *SPL13* are more relevant to both the juvenile-to-adult vegetative transition and shoot growth, while *SPL3/SPL4/SPL5* do not play a major role in vegetative phase change or floral induction, but do promote the floral meristem identity transition through synergistic action with the FT–FD module under long days [31,32]. Expression analysis and transgenic plants for gain-of-function or loss-of-function studies revealed that AtSPL9 promotes flowering by activating floral meristem identity gene *AP1*, whereas AtSPL15 is responsible for floral induction in short days through activation of the MADS-box gene *FUL* and another microRNA gene miR172b, which targets and inhibits several flowering repressor genes such as *AP2*, Schlafmutze (*SMZ*), Schnarchzapfen (*SNZ*), target of EAT1 (*TOE1*), *TOE2*, and *TOE3* [28,33]. Both SPL9 and SPL15 interact with GA signaling protein DELLA and converge with the GA-mediated flowering pathways [34,35,36,37]. Thus, the miR156/SPLs module in *Arabidopsis* represents the endogenous flowering control axis. Nevertheless, fewer studies addressed the molecular pathways controlling flowering induction and competence to flower in most perennials.

Orchids are important horticultural plants with high commercial value. Many orchids have long life cycles, as well as characteristic slow-growing habits, and take more than two years to achieve flowering [38]. Recently, homologs of *FT* and of other floral meristem identity genes were cloned and functionally studied in *Dendrobium* Chao Praya Smile [39], *Oncidium* Gower Ramsey [40], and other orchids [41]. In these studies, *FT* homologs were shown to correlate with flower organ development and accelerated flowering when overexpressed in *Arabidopsis* [40] and Chao Praya Smile [39]. However, whether these *FTs* have a role in regulation of flowering time per se is still elusive, and information regarding the involvement of miR156/SPL in these unique species is lacking. Here, we took advantage of a currently available version of the *Dendrobium catenatum* genome sequence [42] and utilized a genome-mining approach to identify members of both the *FT/TFL1* and the miR156/*SPL*s gene families. The genome of *D. catenatum* encodes nine *FT*/*TFL1* members and 12 *SPL* genes, where seven of the latter are predicted targets of miR156. A BLAST search of the whole-genome sequences resulted in only a single locus of the putative miR156 precursor gene. We further determined the expression profiles of these genes in the three developmental stages: protocorms, one-year-old young (juvenile) plants, and three-year-old (adult) plants. We found in this orchid that a *TFL1*-like gene, *DcHd3b*, is highly expressed in the seedling and juvenile phase, while the *DcSPL3* is predominant in the adult phase.

## 2. Results

### 2.1. Sequence Analysis of FT/TFL1 and SPL Family Members in D. catenatum

**Figure 1:**
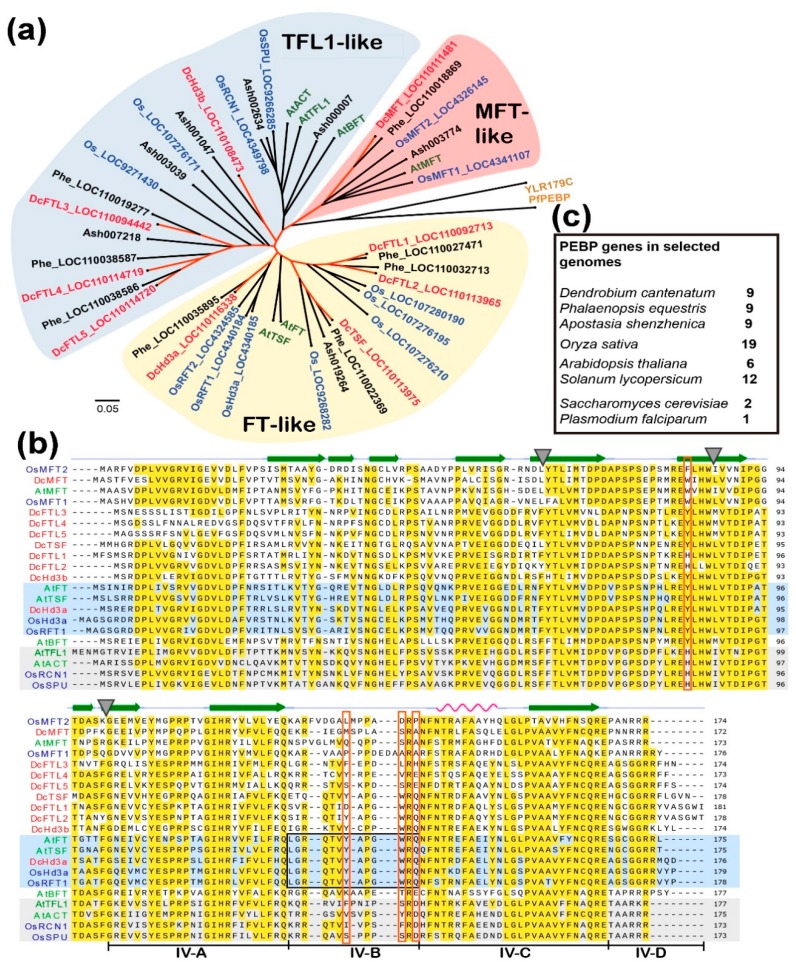
The nine FT/TFL1 proteins encoded by the phosphatidylethanolamine-binding protein (PEBP) gene family in the *Dendrobium cantenatum* genome. (**a**) A phylogenetic tree of FT proteins from *D. cantenatum* (highlighted in red), *Arabidopsis thaliana* (highlighted in green), and other species. Only selected FTs from *Oryza sativa* (Os, in blue), *Phalaenopsis equestris* (Phe), and *Apostasia shenzhenica* (Ash) are included; (**b**) sequence alignment for motif comparisons. The secondary structures are indicated at top of the alignment; the conserved exon boundaries are marked by triangles. The four segments (A, B, C, and D) of the fourth exon important for ligand binding and protein–protein interaction in FT/TFL1, as revealed by Ahn et al., 2006, are line-labeled under the sequences. Known activator FTs and repressor TFL1-like proteins are shaded with light-blue and light-gray, respectively. Red-boxed residues correspond to positions at 85, 134, 138, and 140 for AtFT, and the black-boxed residues denote the highly conserved fragment B in inducer FTs but divergent in the repressor TFLs [7,43]; (**c**) numbers of PEBP genes identified by BLAST survey in some plant species and the single-cell eukaryotes.

To identify genes belonging to the *FT/TFL1* family, we used the AtFT and AtTFL1 sequences to BLAST against the genome of *D. catenatum* and ended up with nine homologous loci, which were annotated in the database either as heading date 3A-like or *FT*-like genes. The deduced amino-acid sequences showed well-conserved PEBP (phosphatidylethanolamine-binding protein) signature motifs and could be assigned within the three subfamilies: *FT*-like, *TFL1*-like, and *MFT*-like with those from *Arabidopsis*, rice, and two other orchid species, *Phalaenopsis equestris* and *Apostasia shenzhenica* (Figure 1a). DcHd3a is the closest ortholog to the founder florigens, OsHd3a and AtFT, with overall amino-acid identities of 78% and 71%, respectively, and it shares 90.91%, 59.88%, and 95.45% identity with the reported OnFT, OnTFL1 [40], and DoFT [39], respectively. DcHd3a also contains the highly conserved amino-acid residues in fragment B, which forms the external loop in the conserved PEBP protein structures as revealed among other inducer FTs [7,9]. Another member, DcHd3b, shares 70% amino-acid identity with DcHd3a. Alignment of these *Dendrobium* sequences with known-function FT/TFL1 orthologous proteins from *Arabidopsis* and rice showed that the four critical amino acids at the positions of Tyr85, Tyr134, Trp138, and Gln140 (numbering according to AtFT sequence) were roughly conserved among *Dendrobium* sequences (Figure 1b). These four residues confer either an inducer or repressor activity of FT/TFL1 [6,7,43]. Similar to the situations in *Arabidopsis* and rice, DcMFT is somehow deviated and separated from the other members of the family (Figure 1a,b). In terms of family size in gnomons, the three orchids, *D. catenatum*, *P. equestris*, and *A. shenzhenica,* contain the same number of *FT/TFL1* genes (Figure 1c).

**Figure 2:**
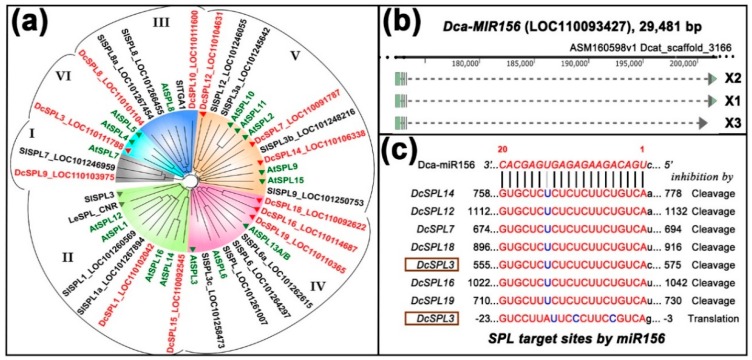
Twelve *SPL* genes and one microRNA (miR)156 gene are found in *Dendrobium catenatum*. (**a**) A phylogenetic tree of SPL proteins from *D. catenatum*, *A. thaliana*, and *S. lycopersicum*. The genome of *D. catenatum* encodes 12 SPLs (highlighted in red), seven of which are predicted targets of miR156 (marked with red triangles). *Arabidopsis* contains 16 SPL proteins (highlighted in green), 10 of which are targeted by miR156 (with green triangles). The tomato genome has more SPL-coding genes than listed here, two of which were experimentally shown to be regulated by miR156 (gray triangles); (**b**) a single locus was identified in the genome which transcribes into three precursor non-coding RNA (ncRNA) variants. Broken lines denote introns, and light-green boxes denote exons; (**c**) miR156-targeted sites in messenger RNAs (mRNAs) of *SPLs*. Note that the *DcSPL3* is possibly inhibited by mRNA cleavage and/or translational blockage.

A homology search with *Arabidopsis* and tomato sequences identified 12 *SPL* genes (annotated as *SPL1*, *3*, *7*, *8, 9*, *10, 12*, *14, 15*, *16*, *18*, and *19*, by the genome project) and one precursor gene of miR156 in *D. catenatum* (Figure 2a,b). We grouped the proteins together with those from *Arabidopsis* and tomato into six clades using alignment with full-length amino-acid sequence, rather than with their nucleotide sequences [44]. The predicted miR156-targeted SPLs could be placed within the IV, V, and VI clades (Figure 2a), suggesting a functional similarity at the protein level. Pairing with the 20-nt sequence of miR156 with its seven target *SPL* transcripts, as predicted by the online tool psRNATarget, http://plantgrn.noble.org/psRNATarget/, revealed only a mismatch in six mRNAs except *DcSPL3,* in which the target sequence exhibited three mismatches, suggesting a repression either by transcription cleavage or translation inhibition, or both (Figure 2c).

In contrast to *Arabidopsis*, where mature miR156 was encoded by eight precursor genes, the genome of *D. catenatum* only encodes a single locus miR156 gene. This gene (LOC110093427) is annotated as uncharacterized and spans a region of 29,481 bp, which produced three non-coding RNA (ncRNA) variants with lengths of 1194 bp, 1332 bp, and 902 bp [42]. Herein, we rename it to *DcMIR156* and its transcription to pre-miRNA156 X1/X2/X3 (Figure 2b). To our knowledge, neither *SPL* nor miR156 were studied in more detail in *Dendrobium* species [45].

### 2.2. DcHd3b Is Highly Expressed in Protocorms and Juvenile Plants

**Figure 3:**
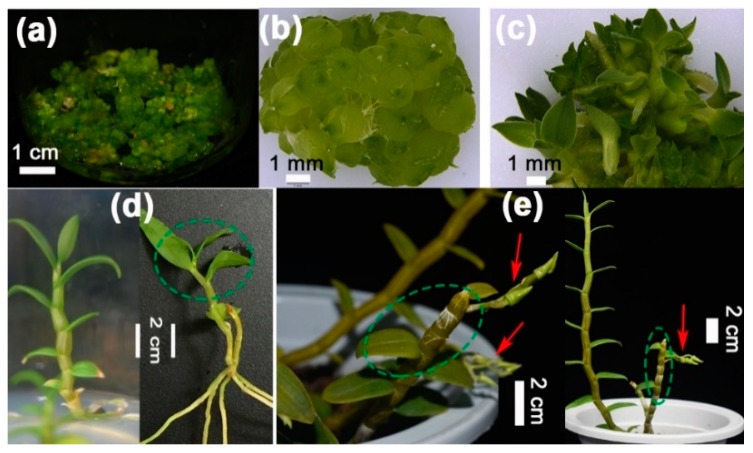
*D. catenatum* plants in the three developmental stages. (**a**) Protocorm clusts on proliferation medium (½ MS + 0.269 μM NAA + 2.22 μM BA + 5% potato pulp + 2.5% sucrose); (**b**) proliferating protocorms on proliferation medium; (**c**) differentiating protocorms at 40 days on germination medium (½ MS +2.69 μM NAA +5% banana pulp + 3% sucrose); (**d**) one-year-old young plants in juvenile stage which were incapable of flowering; (**e**) adult three-year-old plants cultured in pots. Red arrows indicate the lateral floral bud sets. Green circles indicate sampling positions for RNA isolation from leaves and stems.

To determine whether the expression pattern of these genes would follow a developmental stage-specific manner, we isolated total RNA from undifferentiated (Figure 3a,b) and differentiating (Figure 3c) protocorms, leaves, and stems of young juvenile (Figure 3d) or three-year-old adult plants (Figure 3e). Using the 18S RNA as a reference, the relative abundances of mRNAs were detected by quantitative real-time PCR. 

**Figure 4:**
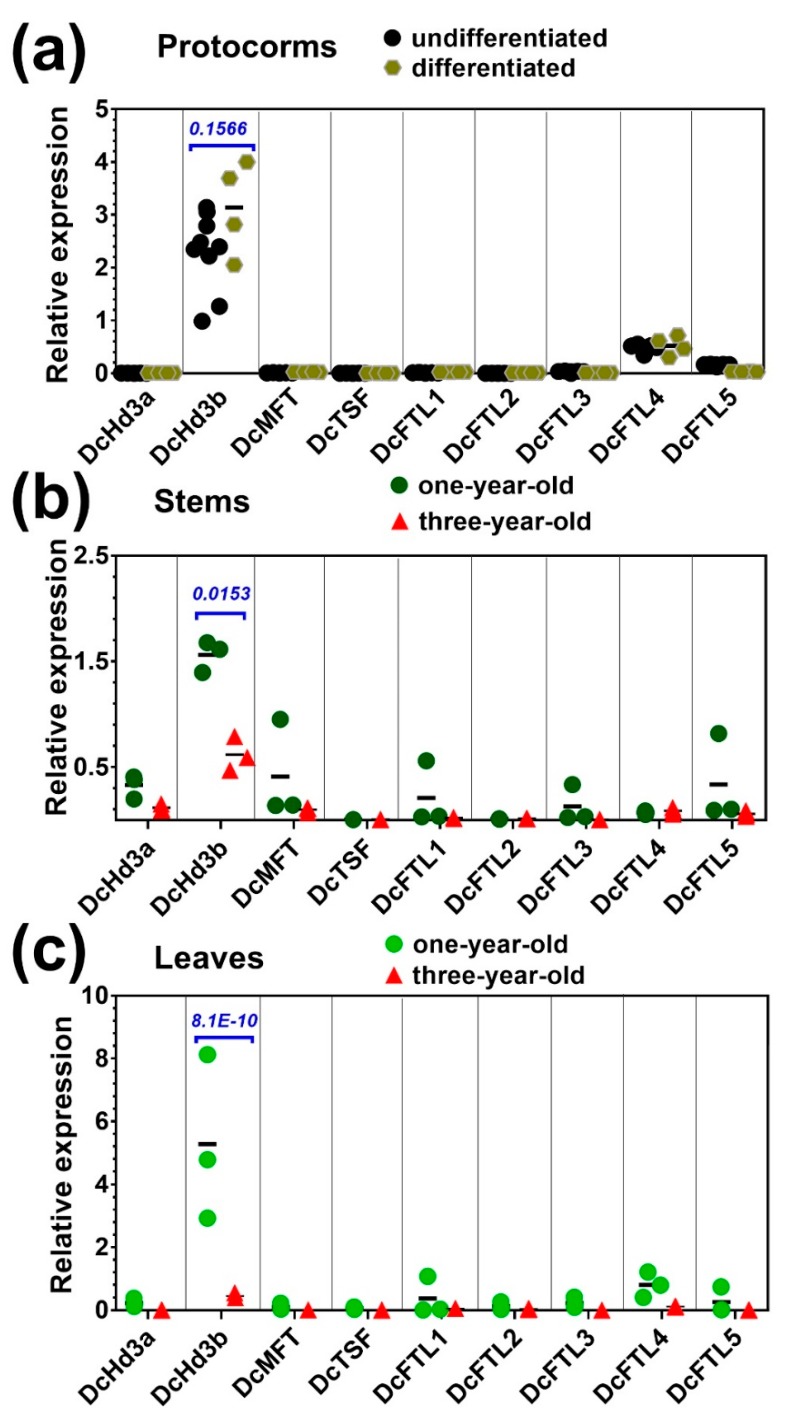
Expression profiles of *FT*/*TFL1* family members in (**a**) protocorms at proliferation and differentiation stages, (**b**) stems of young and adult plants, and (**c**) leaves of young and adult plants. The 18S gene was used a reference. Statistical significance was determined using a paired Student’s *t*-test with alpha = 0.05; the adjusted *p*-value is shown above the data.

Profiling the *FT/TFL1* gene expression showed that, in the protocorms, either undifferentiated or at earlier differentiating stage, *DcHd3b* is the most expressed among the *FT/TFL1* genes, followed by *DcFTL4* (~5.5-fold lower than *DcHd3b*) and *DcFTL5* (~17-fold lower than *DcHd3b*). Transcripts of other *FT/TFL* genes were barely detectable (Figure 4a). No significant change in the expression of *FT*/*TFL1* genes was found during early differentiation of the protocorms, e.g., until leaf primordium opening (Figure 3c). Notably, *DcHd3b* also showed much higher expression in young juvenile plants. Its mRNA levels were approximately 13.5-fold and 2.6-fold higher in leaves and stems of young plants compared to adult plants, respectively (Figure 4b,c). 

Furthermore, *DcMFT*, *DcFTL5*, *DcFTL3*, and *DcFTL1* showed some variation in their mRNA level between young and adult stems, although the difference was not significant, probably due to uneven spatial distributions of these genes in the organs (Figure 4b). Similarly, expression of *DcFTL4*, *DcFTL5*, and *DcFTL1* in leaves between young and adult plants displayed small differences (Figure 4c). Despite no significance, they all seemed to decline with aging. 

Thus, several of the nine *FT*/*TFL1* genes in *D. catenatum*, namely *DcHd3b*, *DcFTL3*, *DcFTL4*, *DcFTL5*, *DcFTL1*, and *DcMFT*, were preferentially expressed in young juvenile tissues or downregulated in adult tissues. *DcHd3b* represented a significantly highly expressed gene in young plants, but was repressed in adult plants.

### 2.3. MiR156 Abundance Correlated Well with Protocorm Germination and Differentiation

**Figure 5:**
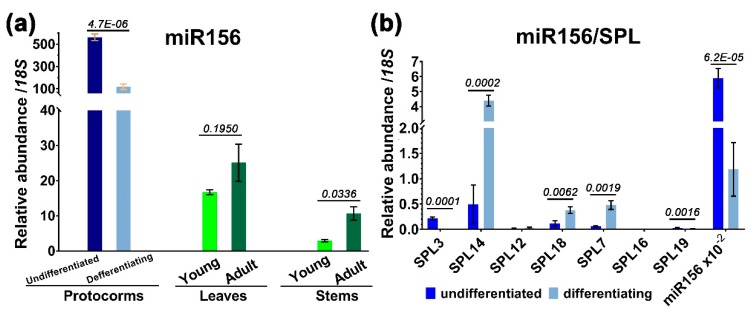
Expression profiles of miR156 in different tissues and of *SPLs* in the protocorms. (**a**) Quantitative stem–loop RT-PCR analysis of miR156 in protocorms, leaves, and stems; (**b**) RT-qPCR analysis of SPL transcript levels in the protocorms. For comparison, a value of 10^−2^ × miR156 was also included. Error bars represent the SD for at least three biological replicates. Statistical significance was determined using a paired Student’s *t*-test with alpha = 0.05; the adjusted *p*-value is shown above the bars.

To determine whether miR156 is expressed differently during development, we used stem–loop quantitative RT-PCR to measure the level of miR156 in protocorms, differentiating protocorms at 40 days, and juvenile and adult plants. The results showed that miR156 was highly accumulated in protocorms, with a level of 2^−Δct^ of 560.52 relative to 18S ribosomal RNA (rRNA), and declined by a factor of ~4.98 at 40 days following germination and differentiation. Mature miR156 level was further reduced by 33.50- and 193.28-fold in one-year-old leaves and stems, and by 22.35- and 52.58-fold in three-year-old leaves and stems, respectively (Figure 5a). The steady-state levels of miR156 of adult plants were slightly higher than in the juvenile plants (not significantly for leaves); however, a tendency to decrease after protocorm differentiation was obvious (Figure 5a).

The decline of miR156 during earlier protocorm differentiation was concurrent with an increase in transcripts of *SPL14*, *SPL7*, and *SPL18*, three of the seven miR156 targets, up to 9.00, 7.85, and 3.45 folds of reduction, respectively (Figure 5b). However, an opposite expression pattern was also observed for *SPL3* and *SPL19* during differentiation, although their levels were rather low (Figure 5b). It is, thus, likely that SPL14/SPL7/SPL18 may be positively involved in the regulatory pathway of protocorm differentiation or seedling development.

Since effective inhibition of miR156 on *SPL* was proposed to require a threshold of at least 100 times greater abundance over its targeted transcripts, as suggested in *Arabidopsis* [27], we compared the abundance of miR156 to the seven *SPL* mRNAs. The ratios were well above this value in undifferentiated protocorms. Even in the differentiating protocorms, two of the three upregulated genes, *SPL18* and *SPL7*, also had an mRNA level of 1/311 and 1/246 of miR156, respectively (Figure 5b). The only exception was *SPL14*, where its transcript was 26.88 times less abundant than miR156. Therefore, at least before the establishment of a seedling, almost all target *SPL* genes were greatly repressed by miR156, leaving the *SPL14* as the least affected gene during seedling development (Figure 5b). 

### 2.4. DcSPL3 Is the Only Significantly Upregulated Gene among the SPL Family in Adult Plants

Although the adult plants did not display a reduced level of mature miR156 in both leaves and stems, as compared with juvenile plants (Figure 5a), a further analysis of the *SPL* expression between juvenile and adult plants would help explore the miR156/SPL module in *Dendrobium* flowering control. 

**Figure 6:**
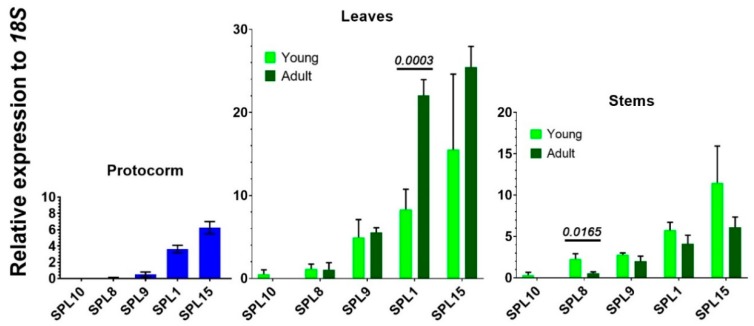
Expression profiles of five *SPLs* that were not targeted by miR156 in protocorms, leaves, and stems. Error bars represent the SD for at least three biological replicates. For statistical significance, the adjusted *p*-values are given on top of the bars.

Among the five SPLs which were not predicted as targets by miR156, transcripts of *SPL15* and *SPL1* were more abundant in all three tissues tested, followed by *SPL9*. Typically, transcripts of these non-target *SPL* were readily detectable, except *SPL10*. Higher expression was mainly found in leaves (Figure 6). With regard to differential expression between young and adult plants, transcripts of *SPL1* were significantly more expressed in adult leaves than in young ones, whereas *SPL8* was preferentially expressed in young stems (Figure 6). 

**Figure 7:**
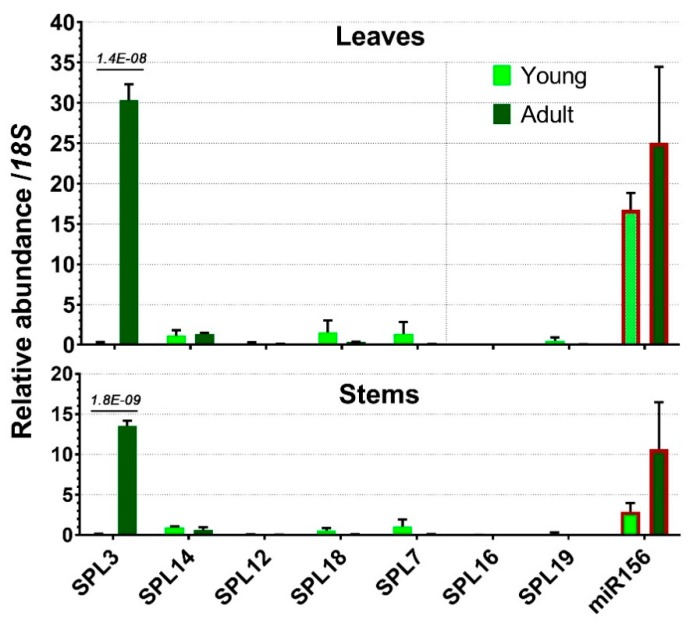
Expression profiles of miR156-targeted *SPL*s in leaves and stems of one-year-old and three-year-old plants compared with miR156 levels as in Figure 5a. Error bars represent the SD of at least three biological replicates. For statistical significance, the adjusted *p*-values are given above the bars.

Except for *SPL3*, all the other targeted *SPLs* displayed very low expression levels in adult leaves or stems (Figure 7). Slightly increased expression in young tissues was observed for *SPL7* and *SPL18*, but these little difference in expression levels could not possibly explain their roles in juvenile phase change.

*SPL3* showed a striking difference in gene expression between adult and juvenile plants. Its transcripts were hardly present in young plants but accumulated to a large amount in both adult stems and leaves, with the latter being the most abundant tissue (Figure 7). Since miR156 in both leaves and stems was not significantly different between young and adult plants, the highly accumulated *SPL3* transcripts in adult plants suggest that *SPL3* mRNA was not sufficiently repressed by miR156 in this stage (Figure 7). Therefore, *SPL3* was the only miR156-targeted gene that was significantly associated with adult plants and was expressed strongly in both leaves and stems.

## 3. Discussion

Time to flower, a process either referring to juvenile–adult phase change or vegetative–reproductive transition, is strictly controlled by an intricate regulatory network involving at least both *FT/TFL1* and the miR156-regulated *SPL* family members. They regulate growth-related downstream genes in response to diverse environmental and developmental signals, and coordinate different flowering pathways to ensure maximal reproductive success [46]. Like most orchid plants, *D. catenatum* requires at least three years from seeds to reach developmental maturation when flowers are induced under favorable environmental conditions. Protocorm, a middle stage of a seedling establishment, might be induced to further differentiation or kept at the proliferation stage (undifferentiated) under in vitro culture. The one-year-old seedlings represent the juvenile stage, and the three-year-old plants are developmentally ready to reproduce. These three stages showed divergent gene expression patterns for both family members for *FT*/*TFL1* and *SPL*.

Most notably, under normal growth conditions in the seedling and juvenile stages, the *TFL1*-like gene *DcHd3b* from the *FT*/*TFL1* family was predominantly expressed (Figure 4), whereas the negative regulatory RNA, miR156, was highly accumulated in seedling but downregulated during further development, which was coincident with the activation of some of its target *SPL* genes: *SPL14*/*SPL7*/*SPL18* in differentiated protocorms and *SPL3* in adult plants (Figure 5, Figure 6 and Figure 7). These results provide hints for further deciphering the mechanism of developmental control and flowering competence in this orchid species. Importantly, verification of their putative functions in controlling flowering time would allow the application of biotechnology to assist molecular breeding of the shortened juvenile phase.

### 3.1. Is there a Repression of Competence to Flower by TFL1-Like Genes in D. catenatum?

The prevailing expression in seedling and young plants but very low detectable transcripts in adult leaves or stems of *DcHd3b* likely implies that a similar mechanism might exist in *D. catenatum*. DcHd3b is closer to the TFL1-like proteins such as terminal flower 1 from *Arabidopsis* and contains a somewhat divergent P-loop region as compared with the floral-promoting FT sequences [43] (Figure 1). Floral-repressing *TFL1* paralogs were also reported in several other plants, for example, in gymnosperm species [10], sugar beet [19], strawberry [47], and rose, in which the *TFL1* paralog *RoKSN* was an intuitive repressor of flowering, while its mutated allele exists in continuous-flowering rose species [44].

It was shown in an orchid plant, *Oncidium* Gower Ramsey, that orthologs of both *FT* and *TFL1* (*OnFT* and *OnTFL1*) were able to complement the respective mutant phenotypes of *Arabidopsis*. Both *OnTFL1* and *OnFT* were expressed highly in pseudobulbs in the reproductive stage and juvenile axillary bud. However, the *OnFT* mRNA was more abundant than *OnTFL1* and it also accumulated in reproductive buds and leaves [40]. In *Dendrobium* Chao Praya Smile, an ortholog of FT was upregulated in reproductive organs, including inflorescence apices, stems, floral buds, and open flowers. Overexpression of *DoFT* promoted flowering and notably pseudobulb formation, while suppressing the endogenous *DoFT* transcripts delayed flowering in the orchid plants [39]. However, these genes seemed to participate in controlling storage or flower organs rather than with juvenile phase transition. Our result of a downregulated expression pattern of *TFL*-like gene(s) in the adult stage suggests that, in *D. catenatum*, there might exist a repressed mechanism to prevent flowering during vegetative growth. However, more work is needed to evaluate the functions of *DcHd3b*.

### 3.2. MiR156/SPL Involvement in Seedling Development and Maturation in D. catenatum

MiR156 is a “negative” regulator in many developmental processes by repressing specific SPL transcription factors at the mRNA level [48,49,50]. The age-dependent miR156-regulated SPL pathway transforms vegetative shoot apical meristem (SAM) into floral bud and control flower development in response to endogenous signals and external hints [22,28,29,31,51]. In the perennial plant *Arabis alpine,* downregulation of miR156 by age and de-repression of its target SPL transcription factors in the apices account for its competence to flower under vernalization [52].

We showed that, during *Dendrobium* protocorm differentiation or early seedling development, the increase in *DcSPL14/DcSPL7/DcSPL18* transcript levels is associated with the reduced abundance of miR156 (Figure 5), indicating an involvement of the three *SPL* genes during these processes. On the other hand, the miR156-targeted *DcSPL3* (the closest homolog of *AtSPL3/4/5* clade) was actively associated with adult stage in both leaves and stems, where its transcript level exceeded that of miR156 (Figure 7). Thus, we speculate that *DcSPL3* might be relevant to the control of plant maturation in *D. catenatum*. 

### 3.3. Is there a Possible Link between the DcHd3b Pathway and the miR156/DcSPL3 Module in Phase Transition in Dendrobium catenatum?

The stage-specific expression patterns of *DcHd3b* and *DcSPL3*, as discussed above, raise an interesting question as to whether and how the two pathways are inter-related. It is well demonstrated in *Arabidopsis* that the FT–FD–14-3-3 protein complex and the downstream gene *SOC1* regulate *SPL3*, *SPL4*, and *SPL5* by directly binding to their promoters in response to photoperiod signals. This regulation is independent of miR156 [13]. In turn, *SPL3/4/5* also directly activated several floral differentiation genes including *SOC1*, Fruitfull (*FUL*), Leafy (*LFY*), Apetala1 (*AP1*), and *FT* [21,22]. Evidences from the perennial *A. alpine* suggested that, in young plants, *TFL1* is required to delay flowering through repression of the floral meristem identity gene *LFY*. Until in the later developmental stage, this blockage of flowering competence was overcome by the upregulated activity of SPL transcription factors which targeted to the common floral meristem identity genes [13,52]. Although not strongly supported yet, a similar mechanism might exist in *D. catenatum*, given that the opposite pattern of expression exists between *DcHd3b* and *DcSPL3* in young and adult. It is, thus, possible that the convergent antagonistic action of both pathways determines the timing of juvenile phase change in *D. catenatum*. Of course, involvements of other members from the *FT*/*TFL* or *SPL* family are not exclusive.

## 4. Materials and Methods

### 4.1. Plant Materials and Growth Conditions

Seeds of *D. catenatum* were germinated aseptically in half-strength MS medium solidified with 3 g/L agar and supplemented with hormones (0.22 μM 6-benzyladenine (BA) + 0.027 μM α-naphthalene acetic acid (NAA) + 0.05 μM indole-3-butyic acid (IBA)). Seed-derived protocorms were proliferated in the liquid proliferation medium (½ MS + 0.269 μM NAA + 2.22 μM BA + 5% potato pulp + 2.5% sucrose) and developed on differentiation medium (½ MS + 2.69 μM NAA + 5% banana pulp + 3% sucrose + 3 g/L agar), both cultured in 25 ± 2 °C, with a 12-h photoperiod and 2000 lux from an LED light source. Seedlings developed from germinating protocorms after ca. four months were grown in pots and maintained in a climate room under long days (14-h light/10-h dark) and 26 °C/22 °C. In our conditions, one-year-old plants were unable to flower; only after three years of growth can plants set flowers. In addition to protocorms at either proliferation or the 40-day differentiation stage, these one-year-old and three-year-old plants were used as materials for RNA isolation.

### 4.2. Gene Identification and Phylogenetic Analysis

The NCBI genome assembly (ASM160598v2) of *D. catenatum* was used for BLAST search homologous sequences of FT/TFL1 and SPL families using known proteins from *Arabidopsis*, rice, and tomato. A BLAST search was also conducted with *Apostasia shenzhenica* [53] and *Phalaenopsis equestris* [54] databases. The mature miR156 20-nt sequence was used to BLAST the *D. catenatum*’s DNA and RNA databases in NCBI to include all putative sequences. All resulting sequences and annotations were manually filtered, selected, and retrieved. For alignments, protein sequences were submitted to MUSCLE (https://www.ebi.ac.uk/Tools/msa/muscle/), and the phylogenetic trees were constructed by MEGA X (https://www.megasoftware.net/) using the maximum-likelihood method with a 1000 bootstrap number. The neighbor-joining trees were generated via Figtree version 1.4.4 downloaded from https://github.com/rambaut/figtree. 

### 4.3. RNA Isolation and Quantitative RT-PCR

For RNA isolation, protocorms, leaves, and stems were collected and frozen in liquid nitrogen. We pooled two plants for leaf and stem samples as one biological replication, and six to 10 protocorms as one. Three biological replications were used. Total RNA was isolated using Trizol (Invitrogen) and treated with DNase (Ambion) following the manufacturer’s instructions. During RNA preparation, approximately 1 g of fresh tissue was ground into fine powder in liquid N_2_, and about 200 mg of the frozen powder was extracted in 500 mL of extraction buffer. Finally, we merged four extractions from the same sample together. These RNA isolations were kept at −80 °C or used immediately for quantitative RT-PCR measurements of miR156 and *FT/TFL1* or *SPL* genes. Usually, 2 μg of total RNA was used in one RT reaction.

To measure the abundance of miR156, the stem–loop qRT-PCR method with SYBR Green was employed as detailed in Reference [55], using an miRNA156-specific RT primer dca-miR156-rt, 5′–GTCGTATCCAGTGCAGGGTCCGAGGTATTCGCACTGGATACGACGTGCTCA–3′ for synthesis of the templates, and the specific qPCR primers dca-miR156-qF, 5′–GCGGCGGTGACAGAAGAGAGT–3′ and dca-miR156-qRV, 5′–GTGCAGGGTCCGAGGT–3′, for quantification. 

For qPCR analysis of transcript levels of *FT*/*TFL1* and *SPL*s, the first-strand complementary DNA (cDNA) was synthesized with random primes using the Prime Script™ First Strand cDNA Synthesis Kit (Takara Biomedical Technology (Beijing, China) Co., Ltd.). Gene-specific primers were designed with the Primer3+ program (http://www.bioinformatics.nl/cgi-bin/primer3plus/primer3plus.cgi) (access on February 20, 2017). and are listed in Table S1 (Supplementary Materials). Specificity of all primer pairs was monitored using the melt curve method to ensure a single peak for each reaction. Before the quantitative PCR, annealing temperature optimization was conducted by testing a range of temperatures from 55 to 70 °C.

All qPCR reactions were performed in triplicate for each biological replicate on a Roche Light Cycler 96, using a PCR program set as an initial step of 30 s at 94 °C, followed by 40 cycles of 15 s at 94 °C, and 30 s at 60 °C. The relative expression levels of genes were calculated by the 2 (−ΔΔ C (T)) method [56], using 18S rRNA as an endogenous reference gene (primers in Table S1, Supplementary Materials).

For statistical analysis, either two-way ANOVA or pair-wide multiple *t*-tests on means of at least three biological replicates was performed using the GraphPad Prism software (version 7). The adjusted *p*-values were presented in the figures.

## Supplementary Materials

The following are available online at https://www.mdpi.com/1422-0067/20/11/2725/s1: Table S1: List of primers used for RT-qPCR.
