# Supplementary material for: Expression Pattern of FT/TFL1 and miR156-Targeted SPL Genes Associated with Developmental Stages in Dendrobium catenatum
Source: Int J Mol Sci. 2019 Jun 3;20(11):2725. doi: 10.3390/ijms20112725 (PMC6600168; doi:10.3390/ijms20112725)
Supplement: Supplementary file 1 [file ijms-20-02725-s001.pdf]

Table S1 List of primers used for RT-qPCR

Gene	Primer sequence 5′-3′
DcHd3a	F TGCAGTATCAAAGGAAGATAGAGATG<br>R CCATTGGTGACATCTTTGGA
DcHd3b	F ATTGGGTTCGGATATTGGAT<br>R GCTCGGTACTCCACAGCATC
DcMFT	F TCTCTATACCTTGATTATGACAG<br>R CAGCCAGTGTATCCATTC
DcTSF	F GTGATGATGAGCAGATTC<br>R CCAGTGTAGGTATTCTCTAT
DcFTL1	F GGATGGCGACAGAACTTCAA<br>R AATCCATCCACTGGCAACAT
DcFTL2	F AAGAATACAACCTTTCCTT<br>R ATCAGTTGTGAAACCTTC
DcFTL3	F ATTGCAGAGCAAAGCATGTA<br>R ATTTTGGAAACCCTCGGAAG
DcFTL4	F GACTGCAACATGCAGATCAA<br>R TGAGACAATAGAGTTGCTGATGA
DcFTL5	F TCGTCACAGCACCTCGTTTA<br>R TCCTTCTCATCACATCCAACA
DcSPL1	F GGTGCTACTACCCCCAGTGA<br>R CATCAGATGAACCAGCGAGA
DcSPL3	F AATGATTCTGCCCACCATGT<br>R AGCAAGTTCGGTCATGAGAGT
DcSPL7	F AACCGTGCTACATCCTTTGC<br>R TCCATTGTTCACTGGAGCTG
DcSPL8	F CACTTTGAGTCGAGCAGCAG<br>R CGTTGTCGATGATGAACCAG
DcSPL9	F CTGCAAGCAATGCTGTTGAT<br>R CAGAAGCTGCAAATGATGGA
DcSPL10	F ACTTTGGGACTGGGACTCCT<br>R GTGGGGGAGGGAAGTAGAAG
DcSPL12	F GGTCATAAAGGCGCCAAAT<br>R AAATCCGAAACTTGCCACTG
DcSPL14	F GAATGCATTGCAGGTGTGTC<br>R CCATCTGGAATTCGATGCTT
DcSPL15	F TTGAGGCAAAATCAGCAGTG<br>R CCTCGGAAGAACAAGCAGAC

DcSPL16	F CTTCCCGTCTGCTCTGTCTC<br>R GGAAATCTGATTCCGTGGAG
DcSPL18	F TCCGCATCTACTCCCTTCTC<br>R TCTTTCGCCAGCACAGTAGA
DcSPL19	F CAGCTAGTTTTGGCCCATCT<br>R CAGCGCAGTGGAGTGTAGTT
Dc18S	F TGAAAATGAGCACCATTGGA<br>R TATAACGCCAGGCCTCAAAC
